# Food Safety in the Age of Climate Change: The Rising Risk of Pesticide Residues and the Role of Sustainable Adsorbent Technologies

**DOI:** 10.3390/foods14213797

**Published:** 2025-11-06

**Authors:** Tamara Lazarević-Pašti, Tamara Tasić, Vedran Milanković, Igor A. Pašti

**Affiliations:** 1 VINČA Institute of Nuclear Sciences—National Institute of the Republic of Serbia, University of Belgrade, Mike Petrovica Alasa 12–14, 11000 Belgrade, Serbia; tamara.tasic@vin.bg.ac.rs (T.T.); vedran.milankovic@vin.bg.ac.rs (V.M.); 2 Faculty of Physical Chemistry, University of Belgrade, Studentski Trg 12–16, 11158 Belgrade, Serbia; igor@ffh.bg.ac.rs; 3 Serbian Academy of Sciences and Arts, Kneza Mihaila 35, 11000 Belgrade, Serbia

**Keywords:** pesticide residues, carbon materials, biosensors, food safety, water contamination

## Abstract

Climate change is increasingly recognized as a critical factor of food contamination risks, particularly through its influence on pesticide behavior and usage. Rising temperatures, altered precipitation patterns, and the proliferation of crop pests are leading to intensified and extended pesticide application across agricultural systems. These shifts increase the likelihood of elevated pesticide residues in food and water and affect their environmental persistence, mobility, and accumulation within the food chain. At the same time, current regulatory frameworks and risk assessment models often fail to account for the synergistic effects of chronic low-dose exposure to multiple residues under climate-stressed conditions. This review provides a multidisciplinary overview of how climate change intensifies the pesticide residue burden in food, emphasizing emerging toxicological concerns and identifying critical gaps in current mitigation strategies. In particular, it examines sustainable adsorbent technologies, primarily carbon-based materials derived from agro-industrial waste, which offer promising potential for removing pesticide residues from water and food matrices, aligning with a circular economy approach. Beyond their technical performance, the real question is whether such materials and the thinking behind them can be meaningfully integrated into next-generation food safety systems that are capable of responding to a rapidly changing world.

## 1. Introduction

Ensuring global food safety in the 21st century is becoming increasingly complex due to the accelerating impacts of climate change. Extreme weather events, rising global temperatures, and shifting precipitation patterns are threatening agricultural productivity. Additionally, they are altering the dynamics of chemical usage and contaminant behavior throughout the food supply chain [1]. Among these, pesticide residues are among the most pervasive and persistent categories of chemical contaminants, with significant implications for human health, food security, and the integrity of ecosystems [2].

Pesticides are widely used to mitigate crop losses caused by pests, diseases, and weeds, and their global application has steadily increased over the past decades [3]. However, climate-induced stressors, such as increased pest pressure, prolonged growing seasons, and the introduction of new invasive species, are driving both the frequency and intensity of pesticide applications [4]. These changes are expected to intensify pesticide loading in agricultural environments, increasing the risk of contamination in crops, soil, and water bodies. Moreover, climate conditions such as temperature, humidity, and soil moisture profoundly influence the fate, transport, and degradation of pesticides, potentially leading to greater residue persistence and accumulation in food commodities [4]. The global mean surface temperature has already risen by approximately 1.1 °C above pre-industrial levels, with projections indicating a further increase of 1.5–2 °C by mid-century under current emission scenarios [5]. These temperature shifts, combined with altered precipitation regimes, humidity patterns, and the growing frequency of extreme weather events, substantially affect pesticide degradation, volatilization, and runoff. Higher temperatures generally accelerate chemical reactions and volatilization [6], whereas intense rainfall and flooding increase leaching and surface transport, ultimately altering the persistence and distribution of pesticide residues in soil, water, and food matrices. Rainfall-driven runoff plays a major role in pesticide transport. Laboratory and field simulations showed cumulative sediment runoff of about 2 tons ha^−1^, with total pesticide losses of 5.7%, 1.4%, and 0.9% of the applied mass for fipronil, clothianidin, and imidacloprid, respectively. Over 2% of the applied pesticides were dissolved in runoff water, while <1.2% were particle-bound. Fipronil exhibited the highest mobility and toxicity potential, exceeding aquatic safety thresholds [7]. Current food safety systems and regulatory frameworks are not fully equipped to address this emerging challenge [8]. Most existing standards, including Maximum Residue Limits (MRLs), are based on static models that do not incorporate climate variability or the cumulative effects of low-dose, chronic exposure to pesticide mixtures. Additionally, traditional risk assessment practices often overlook vulnerable populations and combined toxicological effects, including endocrine disruption and neurotoxicity, which may be amplified under changing environmental conditions [9,10,11].

In response to this growing concern, there is an urgent need to identify and implement effective strategies to mitigate and remove pesticide residues from both food and water systems [12]. Among the most promising solutions are sustainable adsorbent materials, particularly carbon-based sorbents obtained through the thermochemical conversion of agricultural and food processing waste [13]. These materials, such as biochar, activated carbon, and hydrothermal carbons, can be produced from a wide range of biomass precursors, including nutshells [14], fruit peels [15], husks [16], and even spent plant residues from fermentation or distillation processes [17]. Their appeal lies in their abundance, low production cost, tunable surface chemistry, high porosity, and chemical stability under variable environmental conditions [18]. Depending on the synthesis parameters and precursor composition, these materials can exhibit diverse physicochemical properties that influence their affinity toward specific classes of pesticides. What makes biomass-derived carbon materials particularly compelling in the context of climate-resilient food safety is their versatility. They can be incorporated not only into conventional water treatment systems, but also into filtration devices used during food processing [19], rinse solutions [20], smart or active packaging [21], or even edible coatings [22], depending on the regulatory and toxicological profile of the adsorbent. It opens new possibilities for point-of-use or near-source pesticide removal, which is an especially relevant strategy in settings where climate-related disruptions may compromise water quality or increase the baseline pesticide burden in fresh produce. Additionally, because they are derived from agricultural waste, their use supports circular economy principles and offers a low-carbon alternative to conventional synthetic adsorbents [23]. In this light, carbon-based materials are more than remediation tools. They represent a platform for integrating waste valorization, contaminant mitigation, and climate adaptation within a unified framework of food safety.

This review aims to provide a comprehensive synthesis of how climate change affects the occurrence and behavior of pesticide residues, while also highlighting the health risks associated with chronic, low-dose, and mixture exposures. In contrast to most existing reviews that examine occurrence [24,25], toxicology [9], or remediation [26,27] separately, this paper emphasizes their interconnections and situates them within the broader context of climate-driven food safety challenges. Particular attention is given to sustainable carbon-based adsorbent materials derived from agro-industrial residues as remediation tools and as part of a forward-looking framework that integrates waste valorization, contaminant mitigation, and circular economy principles. From this perspective, we aim to connect insights from environmental chemistry, toxicology, materials science, and regulatory policy, with the broader goal of rethinking how pesticide management can adapt to the challenges of a changing climate.

## 2. Climate Change and Pesticide Residue Dynamics in Food and Their Health Implications

**Figure 1:**
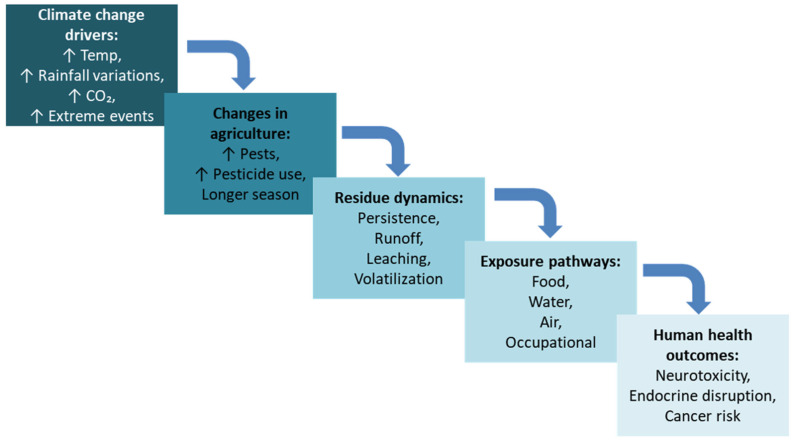
Impact of climate change on pesticide residues and human exposure.

Climate change is reshaping agricultural pest management in ways that directly influence the occurrence, persistence, and distribution of pesticide residues in food. Changes in temperature, precipitation patterns, and atmospheric CO_2_ levels are altering pest pressure, disease cycles, and crop phenology, prompting farmers to adjust both the type and frequency of pesticide applications (Figure 1). Warmer winters can enable pest populations to survive year-round, while prolonged growing seasons may require multiple treatment cycles, resulting in higher cumulative pesticide loads in crops. Beyond application rates, climate variables also affect the environmental fate and transport of pesticides. Elevated temperatures can accelerate volatilization and chemical degradation of certain compounds [28], whereas for others, heat may promote redistribution into plant tissues or soil organic matter [29], thereby extending their persistence. Similarly, altered rainfall patterns influence leaching, runoff, and pesticide migration between soil, water, and crop surfaces [28]. Extreme weather events such as floods can mobilize residues stored in sediments, reintroducing them into agricultural and food production areas [29].

Another factor is the shifting geographic range of pests and crops, which motivates the adoption of pesticides previously uncommon in certain regions. This geographic redistribution often occurs without adequate regulatory adaptation, leading to a mismatch between existing MRLs and emerging contamination patterns. Moreover, climate stress can alter crop physiology in ways that influence pesticide uptake and metabolism, thereby altering residue profiles in the edible parts of the plant [30].

One of the key challenges is the so-called “cocktail effect,” which refers to the co-occurrence of residues from multiple pesticide classes in the same food or water sample [31]. Recent European monitoring reports show that 26% of food samples contain multiple residues, with up to ten co-occurring pesticides detected in surface waters [25,32]. Beyond simultaneous occurrence, field studies indicate that mixture applications can also modify degradation kinetics: the half-life of acetamiprid increased from 4.4 to 9.0 days, while boscalid and pyraclostrobin reached 13.1 and 10.5 days under double-dose and mixture treatments, reflecting the combined effects of co-formulation and environmental conditions such as humidity [33]. Under climate stress, mixtures are more likely to occur, and pesticides may also interact synergistically or additively, increasing their toxicological impact [34]. Current monitoring and risk assessment frameworks rarely capture these combined effects, focusing instead on individual active substances under steady-state conditions [8]. These dynamics suggest that climate change is not simply adding variability to an already complex system. In fact, it systematically shifts the baseline, challenging the assumptions underlying current food safety monitoring and residue regulation. Understanding these interactions is therefore crucial for designing effective mitigation strategies that remain viable under future climate scenarios.

The changes in pesticide application patterns and environmental behavior driven by climate variability have direct consequences for human health. Notably, both chronic low-dose exposure and occasional acute exposure remain major concerns [35]. Chronic exposure, even at levels below current regulatory limits, has been linked to a variety of long-term effects, including neurotoxicity [36], endocrine disruption [37], metabolic alterations [38], and, in some cases, carcinogenicity [39,40]. Acute exposure, although less frequent, can result in immediate toxic effects, particularly among agricultural workers or populations residing in regions with high pesticide use [41]. Vulnerable populations, such as children, pregnant women, and the elderly, are especially at risk [42,43]. For example, developing nervous systems are susceptible to organophosphates [44], while endocrine-disrupting compounds can interfere with hormonal regulation during critical stages of development [45]. Addressing these challenges requires improved analytical capabilities for detecting multiple residues simultaneously, as well as the development of mitigation strategies that can reduce exposure at various points in the food chain.

## 3. Current Strategies for Pesticide Removal

**Figure 2:**
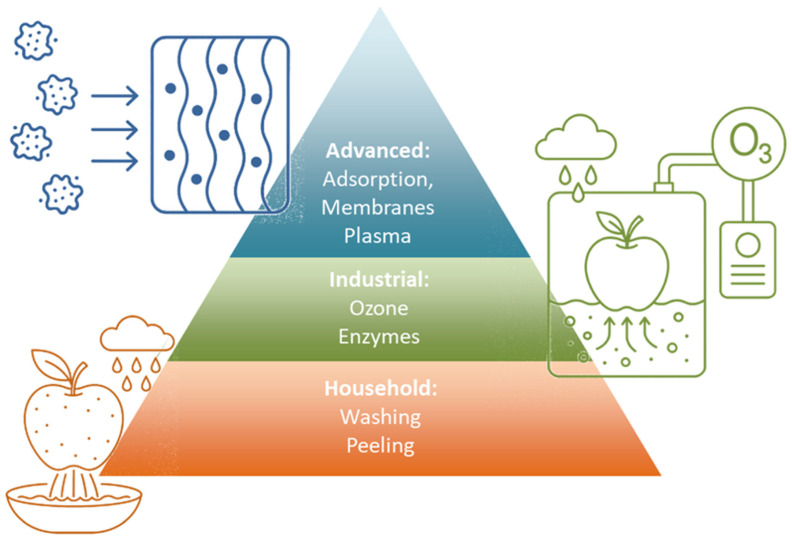
Pesticide removal strategies.

A variety of approaches have been developed to reduce pesticide residues in food and water, ranging from relatively simple household methods to advanced engineering solutions applied in industrial or environmental settings (Figure 2). These strategies differ in efficiency, cost, and applicability, and their relevance is often context-dependent.

Conventional food processing methods have been used for a long time. Physical and mechanical methods are often the first line of defense. Washing, peeling, and cooking can lower pesticide residues on fruits and vegetables, though their effectiveness depends strongly on the chemical properties of the compound. For example, hydrophilic pesticides are more easily reduced by washing [46], whereas systemic or lipophilic pesticides tend to persist in plant tissues [47,48]. Thermal processing may degrade some residues but can also generate transformation products whose toxicological significance is not always well understood [49]. In their study, Flamminii et al. [50] investigated the effects of typical household and industrial processing steps, such as washing, blanching, freezing, and frozen storage, on pesticide residues in spinach. The authors monitored four pesticides (propamocarb, lambda-cyhalothrin, fluopicolide, and chlorantraniliprole) and the degradation product propamocarb n-desmethyl. They showed that washing reduced fluopicolide and chlorantraniliprole levels by 40–47%, while 2 min blanching further decreased residue concentrations, with reductions ranging from 4% to 41%. Longer blanching times showed variable effects, particularly for propamocarb, which reached a 56% reduction after 10 min. Frozen storage led to slight increases in pesticide residues after prolonged blanching. The authors concluded that a 2 min blanching at 80 °C, followed by freezing, was the most effective compromise for reducing pesticide residues in spinach. Wu et al. [51] compared the effectiveness of various home and commercial washing methods for removing 10 common pesticide residues from kumquat, cucumber, and spinach. They investigated the influence of tap water, micron calcium solution, alkaline electrolyzed water, ozone water, active oxygen, and sodium bicarbonate. The results showed that washing kumquats and cucumbers with alkaline electrolyzed water, micron calcium, and active oxygen solutions achieved the best removal rates. In contrast, in spinach, sodium bicarbonate, ozone water, and active oxygen were found to be more effective. Active oxygen solution consistently demonstrated superior efficiency due to its combined alkalinity and oxidizing capacity. Pesticide removal generally improved with increased washing time. Pyrethroid pesticides were the easiest to remove, whereas chlorpyrifos proved the most resistant to washing.

Chemical treatments such as ozonation, chlorine-based washing, or mild oxidants are applied at both household and industrial scales. Ozone is particularly effective against a wide spectrum of pesticides, but its application requires careful control to avoid secondary by-products. In fresh-cut onions, ozonated water washing for up to 5 min effectively reduced residues of organophosphates and carbamates, such as chlorpyrifos and methomyl, more than water washing [52]. Comparable effects were observed in table grapes, where storage under ozone-enriched atmospheres accelerated the degradation of certain fungicides, particularly azoxystrobin, without altering key physicochemical parameters. However, a slight increase in weight loss was noted [53]. Carrots treated with gaseous or aqueous ozone also showed remarkable reductions in difenoconazole and linuron, with removal efficiencies exceeding 80% after two hours of treatment and surpassing 95% after storage, without detectable formation of toxic by-products [54]. Similarly, aqueous ozone treatment of fresh-cut cabbage significantly reduced residues of commonly used pesticides, suppressed microbial growth, and prolonged shelf life [55]. Finally, application of ozonized water through household food purifiers demonstrated substantial reductions in pesticide residues in okra and green chili, particularly for acetamiprid and ethion, thereby lowering consumer exposure risks [56].

Similarly, chlorine can remove certain pesticide residues, but it also raises concerns about disinfection by-products and consumer acceptance. Chlorine dioxide treatments have been shown to significantly reduce residues of phorate and diazinon on fresh lettuce, achieving degradation rates up to 80% in aqueous solutions. The effectiveness of chlorine dioxide depended on multiple factors, including concentration, pH, treatment time, and the chemical nature of the pesticide, with phorate generally more susceptible to oxidation than diazinon. Analysis of degradation products confirmed the formation of oxidized metabolites, supporting the use of chlorine dioxide as a safe and targeted approach for residue reduction [57]. Chlorine washes have also been applied to fruit surfaces with comparable success. In apples, chlorine solutions reduced residues of azinphos-methyl, captan, and formetanate-HCl by 50–100%, with higher pH and temperature enhancing degradation rates [58].

Kitchen-scale electrolyzed water devices (EWDs) represent another promising approach for household-level decontamination. Studies on lemons, cucumbers, and carrots have demonstrated that EWDs can remove up to 80% of water-soluble pesticides, such as malathion and fenitrothion. However, lipophilic compounds like DDT were less effectively removed (20–40%) [59]. While traditional washing methods sometimes outperformed EWDs for certain lipophilic pesticides, these devices offer a convenient, low-chemical alternative for reducing consumer exposure to more soluble contaminants.

Biological methods for pesticide removal rely on microorganisms or enzymes capable of degrading pesticides. Recent studies emphasize the beneficial role of microbial biofilms in bioremediation. Bioremediation has been widely investigated for contaminated soils and wastewater, though its application to food matrices is less straightforward. Biofilm-forming bacteria such as *Bacillus*, *Pseudomonas*, and *Lactobacillus* sp. produce extracellular polymeric substances that enhance microbial adhesion, resilience, and pollutant degradation under adverse environmental conditions [60]. *Pseudomonas* sp. achieved 90.9% chlorpyrifos removal within 120 h in the presence of ZnO nanoparticles [61]. However, biodegradation does not always imply detoxification. During *Bacillus* sp.-mediated malathion degradation, GC-MS analysis revealed the formation of malaoxon, a metabolite considerably more toxic than malathion itself, indicating the need for cautious interpretation of biodegradation efficiency [62]. Enzymatic treatments show promise for the selective degradation of specific pesticide classes, such as organophosphates, but scalability and regulatory approval remain challenges. Bio-enzyme cleaning agents, based on *Aspergillus niger* J6 enzymes, have demonstrated remarkable efficiency in degrading organophosphate residues on produce surfaces. Reported removal rates for common pesticides such as omethoate, phorate, phoxim, and parathion-methyl reach up to 98.5%, indicating that enzymatic treatment can act rapidly and selectively without the use of harsh chemicals. The formulation typically combines the enzyme liquid with stabilizers and absorbents, ensuring practical applicability in food processing or household decontamination [63]. Beyond surface treatments, immobilized enzymes offer promising solutions for water and wastewater remediation. Organophosphorus acid anhydrolase encapsulated in alginate beads has been shown to efficiently hydrolyze ethyl paraoxon in contaminated water, achieving 87–97% degradation in lab-scale continuous systems. Immobilization enhances enzyme stability, allowing for repeated use over multiple batch cycles and maintaining substantial activity over extended storage periods [64].

Advanced technologies include adsorption using activated carbon and other porous materials, photocatalysis, membrane filtration, and advanced oxidation processes (AOPs). These methods are primarily applied at the water-treatment level but are also increasingly considered in food safety contexts. Adsorption, in particular, is attractive because it can be tuned through material design and does not generate harmful by-products, although regeneration of adsorbents can be a limiting factor. Viscose-derived activated carbon fibers have demonstrated remarkable adsorption capacities for organophosphates, such as malathion and chlorpyrifos. By carefully tuning properties such as surface area, pore volume, and pore size, these materials can achieve fast and selective adsorption, reaching equilibrium within minutes. Their application in liquid food matrices, including lemon juice and mint ethanol extracts, allows for high contaminant removal without compromising nutritional or organoleptic quality, and the materials can be regenerated through multiple cycles with minimal performance loss [19]. Non-thermal plasma technologies have also shown significant promise. Plasma needles and related systems effectively degrade pesticides such as dimethoate in water, producing transient oxidation products, such as omethoate, which are subsequently removed, ultimately reducing the overall toxicity of the treated solutions. Adjustments in treatment parameters, including the addition of radical promoters like hydrogen peroxide, can further enhance degradation efficiency. Plasma-based methods are particularly appealing for their low energy requirements, minimal waste generation, and adaptability to real samples [65].

The emerging consensus from multiple studies indicates that combining advanced techniques can further improve pesticide removal outcomes [66]. Synergistic effects are observed when treatments such as ultrasound, ozone, electrolyzed water, ultraviolet radiation, and plasma irradiation are combined with each other or with conventional processing methods, such as washing and blanching. The combination of physical and chemical mechanisms leads to higher removal efficiencies, reduced variability, and improved consistency across diverse food matrices. For instance, ozone applied in combination with microbubbles, lactic acid, or UV irradiation has been effective in reducing residues in vegetables such as baby cabbage, tomatoes, cucumbers, and lettuce [66,67]. Also, ozone-based advanced oxidative processes combining O_3_ and UV irradiation achieved nearly complete (up to 97%) removal of pesticide residues in dried peppers, while preserving capsaicinoid levels, color, and lipid stability [68]. Similarly, ultrasound appears particularly versatile as a co-treatment, amplifying the effects of other technologies [69].

Although the range of available strategies for pesticide removal is broad, none of them provides a universal solution. Conventional food-processing methods are inexpensive and consumer-friendly, but they are limited in effectiveness against systemic pesticides. Chemical treatments, while powerful, raise concerns about by-product formation and safety. Biological approaches demonstrate selectivity but are limited to specific compounds and are often difficult to scale. Advanced physicochemical methods such as adsorption or plasma irradiation offer high efficiency, yet their complexity, cost, and regeneration requirements prevent widespread application. What becomes clear is that these methods should not be viewed in isolation but rather as complementary measures. Combining household practices with industrial-scale treatments and pairing conventional steps with novel technologies can enhance overall efficiency and reliability. More importantly, any technical solution must be supported by preventive agricultural practices and robust regulatory monitoring, since remediation at the consumer or processing stage can only partially mitigate pesticide exposure. In this sense, removal strategies should be seen as one layer within a broader, multi-level system of food safety, rather than as definitive endpoints [8].

Among the advanced removal strategies, adsorbent-based techniques have attracted particular attention due to their versatility, adaptable surface chemistry, and potential for regeneration [70]. In recent years, the growing emphasis on circular-economy principles and green material design has positioned sustainable adsorbents as more than just a technical solution. Moreover, they represent a bridge between environmental remediation and resource recovery. For this reason, these materials warrant dedicated discussion in the following section, focusing on their composition, mechanisms, and sustainability advantages.

## 4. Sustainable Adsorbent Materials for Mitigating Pesticide Residues

The use of sustainable adsorbent materials has emerged as a promising strategy to reduce pesticide residues in both food and water. Carbon-based materials, including biochar, activated carbon, and hydrothermally derived carbons, have attracted particular attention due to their large surface areas, tunable porosity, and versatile surface chemistry, all of which can be optimized for the effective capture of pesticide molecules. In food systems, these materials can help remove residual pesticides from fresh produce, fruit juices, or liquid extracts, while in water, they provide an efficient barrier against contamination of drinking or irrigation supplies. A key advantage of these adsorbents is the potential to derive them from agro-industrial residues such as fruit pomace, nut shells, husks, and other processing by-products. Using such waste as precursors lowers production costs and supports circular-economy principles by transforming agricultural waste into functional materials. This dual benefit aligns with sustainability goals: reducing pesticide contamination while minimizing environmental impacts from waste disposal [71].

**Figure 3:**
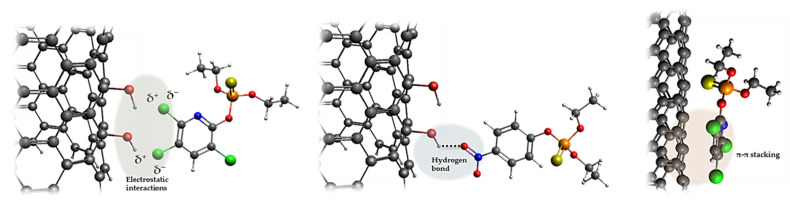
Examples of pesticides’ interactions with carbon surfaces. Atoms are color-coded as follows: grey-carbon, white-hydrogen, green-chlorine, blue-nitrogen, red-oxygen, orange-phosphorus, and yellow-sulfur.

Pesticide removal by carbon-based adsorbents relies on multiple mechanisms (Figure 3). π-π interactions between aromatic structures in the pesticide and graphitic domains of the carbon are often dominant [72], while hydrophobic interactions favor nonpolar pesticide adsorption [73]. Hydrogen bonding and electrostatic interactions can further enhance binding for polar or ionizable compounds [74,75]. Material properties, such as surface functional groups, pore structure, pH, and temperature, significantly influence adsorption efficiency and selectivity, whether the target is a pesticide in water or bound residues on fruits and vegetables [76].

Beyond direct remediation, sustainable adsorbents can be integrated into food-related applications. For instance, incorporating activated carbon or biochar into packaging materials may help reduce residual pesticides on fresh produce during storage, offering a proactive approach to food safety [21,77,78]. In water systems, these materials can be used in filtration or treatment setups to reduce pesticide concentrations, thereby preventing contamination of drinking water or irrigation supplies [77,79].

Nowadays, research on sustainable carbon-based materials for the removal of pesticides from water is among the most prevalent. Table 1 presents the most recent studies addressing this topic.

Sustainable adsorbent materials present a versatile and eco-friendly approach to mitigating pesticide residues in both food and water. Their combination of renewable precursors, tunable adsorption properties, and adaptability for integration into food safety or water treatment systems highlights their potential to become key components of future contamination management strategies. Across all material types, key operational parameters such as pH, temperature, porosity, and surface chemistry significantly influence adsorption efficiency. The choice of precursor, activation method, and functionalization strategy determines both the adsorbent’s capacity and selectivity, underscoring the importance of material design for effective and sustainable pesticide removal. Integrating these materials into food processing steps, filtration systems, or active packaging could provide practical, low-cost, and environmentally compatible solutions to mitigate pesticide exposure in the food supply chain, aligning with the principles of circular economy and climate-resilient food safety strategies. While sustainable adsorbent materials offer an attractive route to reduce pesticide residues, translating laboratory successes into practical applications requires addressing challenges such as raw material variability, stability under diverse food and water conditions, and regulatory approval. Future progress will depend on coupling material design with toxicological validation and cost-effective scaling, ensuring that circular economy principles translate into realistic, climate-resilient food safety solutions [93].

Ensuring the safety and post-use management of adsorbent materials is essential for their realistic deployment. Before integration into food-contact or water-treatment systems, each carbon material must be evaluated for potential leaching of soluble organic compounds or nanoparticles to avoid secondary contamination. Several studies have confirmed that well-stabilized biochars and activated carbons exhibit negligible leaching under neutral conditions [94,95,96]. However, performance may vary depending on precursor purity and surface modification. Equally important is the regeneration and end-of-life handling of pesticide-loaded adsorbents. Thermal or solvent regeneration can recover adsorption capacity, yet repeated cycles may lead to carbon structure degradation or desorption of toxic intermediates [97,98]. When regeneration is not feasible, controlled incineration or encapsulation in inert matrices is recommended to prevent re-release of bound pesticides, ensuring safe disposal and alignment with circular-economy principles [99,100].

## 5. Analytical Methods for Detecting Pesticide Residues in Food and Water

Reliable detection of pesticide residues is a crucial component of ensuring food safety, public health, and environmental protection. Analytical methods have advanced significantly in recent decades, moving from conventional laboratory-based chromatographic techniques to more rapid and field-deployable spectroscopic tools and biosensors. Each method has distinct advantages and limitations in terms of sensitivity, selectivity, cost, and applicability under real-world conditions [101]. A comparative overview is provided in Table 2, while representative applications are summarized in subsequent tables. Although chromatographic and mass spectrometric platforms remain the standard for pesticide residue determination, several alternative analytical methodologies, such as molecular fluorescence for sulfonylurea herbicides, have been explored for specific compounds, providing rapid and solvent-efficient detection in certain matrices [102].

### 5.1. Chromatographic Techniques

Chromatographic techniques, particularly high-performance liquid chromatography (HPLC) and gas chromatography (GC), as well as their coupling with mass spectrometry (LC-MS/MS and GC-MS), remain the standard for regulatory compliance. These methods provide high precision, low detection limits, and the ability to analyze multiple residues simultaneously [104,108]. However, chromatographic approaches are cost- and labor-intensive, requiring sophisticated instrumentation, trained personnel, and elaborate sample preparation [109,110]. Their reliance on centralized laboratories limits broader accessibility, particularly in low-resource or climate-stressed regions [110]. Still, they continue to provide the reference framework against which other methods are validated. Table 3 contains representative studies in which chromatographic methods have been successfully applied for detecting pesticides in a wide range of foods, including vegetables, cereals, honey, meat, and aquatic products. The diversity of analytes, detection ranges, and recovery rates reflects both the power and flexibility of chromatographic platforms. At the same time, it emphasizes the challenges of adapting them to routine or field-based monitoring. For example, reported limits of detection are typically in the ng/g to μg/L range, with recoveries frequently above 80–100%, showing the robustness and reliability of these methods. Chromatography, therefore, remains indispensable for regulatory monitoring and confirmatory analysis, even though its cost and infrastructural requirements limit broader accessibility [111]. Sample extraction and cleanup methods, such as QuEChERS, solid-phase extraction (SPE), and dispersive SPE (d-SPE), are routinely employed prior to chromatographic analysis to ensure matrix removal and compliance with regulatory standards.

### 5.2. Spectroscopic Methods

Spectroscopic methods, including infrared (IR), Raman, UV–Vis, and, more recently, surface-enhanced Raman scattering (SERS), have emerged as rapid and non-destructive tools for detecting pesticide residues. Their key advantages lie in the minimal sample preparation and suitability for high-throughput screening [103], which makes them particularly attractive for quality control and preliminary monitoring in food supply chains. Despite these benefits, spectroscopic approaches generally exhibit lower sensitivity and selectivity compared to chromatographic techniques [127] and are more susceptible to matrix interferences, which restricts their use for regulatory compliance [128]. Nevertheless, they provide valuable complementary information and hold promise for integration into portable or field-deployable devices.

Table 4 presents examples of SERS- and Surface Plasmon Resonance (SPR) based applications in detecting pesticides in fruits, vegetables, dairy products, and beverages. These approaches demonstrate their potential for integration into portable devices, providing rapid results in the field. Still, reproducibility and robustness under variable environmental conditions remain critical challenges for the broader adoption of these technologies. Reported recoveries for spectroscopic methods typically fall within the 85–120% range, which shows their potential reliability. However, results often vary depending on matrix complexity and environmental conditions. Taken together, spectroscopic methods are best positioned as fast pre-screening techniques in production and quality control settings, while chromatography remains indispensable for confirmatory analysis.

### 5.3. Rapid and Field-Deployable Methods

Field-deployable methods, including biosensors, immunoassays, and paper-based devices, have attracted increasing attention as tools for decentralized and accessible pesticide residue monitoring. Their main advantages are rapid and user-friendly operation, often providing results within minutes, and the ability to be applied by farmers, regulators, or food processors without specialized training. Their portability makes them particularly useful in regions with limited laboratory infrastructure or in climate-stressed environments where timely detection is essential. Despite these strengths, the performance of field-deployable methods remains constrained by issues of accuracy, stability, and reproducibility, as well as the need for calibration against reference chromatographic techniques. Stability under variable storage and environmental conditions remains a key barrier to broader adoption.

Table 5 summarizes representative immunoassays applied in different food and water matrices. For example, ELISA enabled triazophos detection in water and apple juice at ng/mL levels [141]. Detection limits for these methods typically range from ng/mL to pM, placing them among the most sensitive portable tools available.

Table 6 highlights examples of biosensor platforms and paper-based devices that rely on colorimetric and fluorescent detection principles. Such tools are especially promising for decentralized, real-time monitoring of pesticide residues.

Analytical methods for pesticide residue detection exist along a continuum from highly sensitive but resource-intensive laboratory platforms to portable, rapid, and accessible field tools. In the era of climate change and growing food safety challenges, striking a balance between accuracy and accessibility is crucial. Chromatographic techniques will continue to serve as the backbone for confirmatory and regulatory analysis, while spectroscopic and biosensor-based approaches can expand monitoring capacity by providing rapid, on-site screening. The most promising path forward lies in combining these complementary approaches, leveraging laboratory precision with field-based flexibility to build food safety systems that are both reliable and resilient.

## 6. Future Perspectives and Research Needs

**Figure 4:**
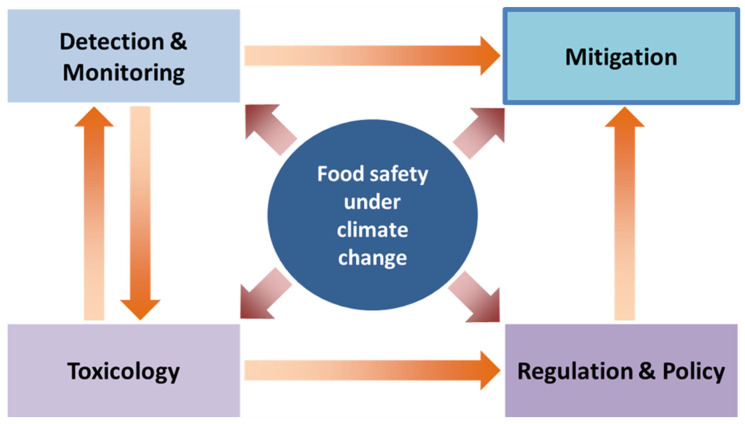
Toward climate-resilient food safety systems. Detection and monitoring, toxicology, regulation and policy, and mitigation are mutually linked, illustrating that effective food safety management requires an integrated approach combining scientific assessment, policy action, and adaptive risk control.

The relationship between climate change, food safety, and pesticide residues requires a holistic, forward-looking approach that integrates detection, removal, and toxicological evaluation into a unified framework (Figure 4). Existing systems are often fragmented. Monitoring is separated from mitigation, while toxicological assessment remains disconnected from material design and regulatory adaptation. Future research should therefore aim to close these gaps and promote cross-disciplinary innovation. One key direction is integrating climate-resilient and circular-economy principles into food safety strategies [160]. It means designing adsorbent materials that are robust across various environmental conditions (extreme temperatures, fluctuating pH levels, or complex food and water matrices), while maintaining high efficiency in laboratory conditions. Agro-industrial waste streams provide a vast and underutilized resource for developing such materials, enabling dual benefits of waste valorization and pesticide remediation [161]. Research should also explore hybrid systems that combine carbon-based adsorbents with catalytic or biological functionalities. In that way, multifunctional platforms capable of both capturing and degrading pesticide residues could be created. Another priority is the development of smart detection and monitoring tools that can be integrated into real-time food safety systems. Biosensors, portable spectroscopic devices, and advanced data analytics powered by artificial intelligence could provide early-warning systems [162], particularly valuable in regions most vulnerable to climate-driven pest expansion. Coupling these detection tools with on-site mitigation strategies, such as adsorbent-based filters or active packaging, would allow for proactive management of contamination risks.

From a regulatory perspective, there is an urgent need to move beyond static MRLs [8]. New frameworks must incorporate climate variability, cumulative exposures, and mixture effects (“cocktail effect”), which are increasingly predominant under climate stress. International harmonization of standards, combined with region-specific adaptation, will be essential to manage pesticide risks in a globalized food supply chain.

A crucial dimension of future progress lies in strengthening interdisciplinary collaboration. The challenges at the interface of climate change, food safety, and pesticide residues cannot be adequately addressed within the boundaries of a single discipline. Chemists and materials scientists can provide advanced sorbents with tailored surface properties, toxicologists can clarify the health implications of chronic low-dose exposures, and food technologists can identify realistic pathways for integrating new materials into processing chains. At the same time, policymakers and regulatory bodies play a decisive role in ensuring that scientific advances are translated into standards, guidelines, and practices that safeguard both human health and the environment. Only by developing genuine dialog across these communities can food safety systems evolve into resilient, adaptive frameworks. In this sense, the goal is not simply to develop more efficient remediation technologies, but to embed them within a broader vision in which environmental protection, sustainable resource use, and public health are mutually reinforcing rather than competing priorities.

## 7. Conclusions

This review demonstrates that climate change contributes to increased variability in pesticide behavior in food systems and also significantly alters the conditions under which these chemicals are used, transported, and accumulated. Such shifts pose significant challenges for both public health protection and the integrity of regulatory frameworks. Current monitoring practices, still primarily focused on single-compound assessments under stable conditions, are increasingly inadequate in capturing the complexity of chronic, low-dose, and mixture exposures that characterize food systems under climate stress. Sustainable carbon-based adsorbents derived from agro-industrial residues offer a promising pathway to mitigate these risks. Their appeal lies in their technical performance, and also in their capacity to connect waste valorization with pesticide removal. In this way, food safety and environmental sustainability are united within a single framework. Still, their adoption cannot be seen in isolation. They must be embedded into a broader system that includes advanced detection methods, preventive agricultural practices, and regulatory reforms that account for climate variability and cumulative exposures. What emerges from this synthesis is the recognition that food safety in the era of climate change cannot be treated as a narrow technical challenge. It requires a rethinking of priorities and a willingness to build bridges between disciplines that do not traditionally collaborate. The future of pesticide residue management will depend on whether we succeed in creating food safety systems that are scientifically robust and socially and environmentally responsive. Achieving this will demand persistence, creativity, and above all, a shared commitment to ensuring that human health and ecological integrity remain central to agricultural progress.

## Figures and Tables

**Table 1:** Summary of carbon materials derived from agro-industrial precursors for pesticide removal.

Agro-Industrial Precursor	Synthesis Conditions	Pesticide	Adsorption Capacity (mg/g)	Specific Surface Area (m^2^/g)	Reference
Rice straw	Dried (60 °C, 24 h, <10% moisture), cut (<5 cm), pyrolyzed at 600 °C (N_2_, 3 °C min^−1^, 1 h); ground, sieved (60 BSS), stored in PTFE.	Atrazine	838.1	220.2	[80]
		Imidacloprid	852.3		
Olive kernel	Dried, milled (1 mm), oven-dried (105 °C, 24 h); pyrolyzed at 800 °C (N_2_, 27 °C min^−1^, 1 h); steam-activated (800 °C, 30 min, 0.5 bar).	Bromopropylate	16.88	600	[81]
Corn cobs			26.64	630	
Rapeseed stalks			106.29	490	
Soya stalks			59.44	570	
Spent coffee grounds	Washed, dried (RT, 24 h), carbonized at 900 °C (N_2_, 5 °C min^−1^, 1 h); impregnated with H_3_PO_4_ (1:2), re-carbonized (900 °C, 1 h); washed (NaOH, water, EtOH), dried, stored	Malathion	92.0	846	[82]
		Chlorpirifos	259		
Spent coffee grounds	Washed, dried (RT, 24 h), carbonized at 400 °C (N_2_, 100 L h^−1^, 1 h). activation: chemical (KOH or H_3_PO_4_, 1 mol dm^−3^, 2:1), physical (CO_2_), or combined activation.	Malathion	11.2	6	[83]
		Chlorpirifos	16.1		
Fig pomace	Dried, pyrolyzed at 500 °C (N_2_, 5 °C min^−1^, 2 h); acidified (HCl, pH 5.0), dried (60 °C, 24 h); modified by γ-irradiation (Co^60^, 50 kGy, 8 kGy h^−1^).	Malathion	0.625	-	[84]
		Chlorpyrifos	0.495		
Plum pomace	Dried, pyrolyzed at 500 °C (N_2_, 5 °C min^−1^, 2 h); acidified (HCl, pH 5.0), dried (60 °C, 24 h); modified by γ-irradiation (Co^60^, 50 kGy, 8 kGy h^−1^).	Malathion	1.067	-	[85]
		Chlorpyrifos	0.219		
Pine bark	Ground (1–2 mm), washed, dried (30 °C, 24 h). Modified with NaOH (2.5 mol dm^−3^), HNO_3_ (1 mol dm^−3^), or H_2_O_2_ (1 mol dm^−3^) (180 rpm, 24 h, 25 °C); washed, dried (50 °C, 48 h). HTC hydrochar: 220 °C, 12 h, 25% solid slurry.	Atrazine	0.522	-	[86]
Corn cob	Sun-dried (1 week), oven-dried (105 °C, 24 h), pyrolyzed 400–600 °C (O_2_-limited, 3 °C min^−1^, 2–6 h).	Atrazine	19.58	303.36	[87]
Tea waste	Air-dried, ground (<1 mm), pyrolyzed at 700 °C (N_2_, 7 °C min^−1^, 2 h). With steam activation (5 mL min^−1^ steam, last 45 min).	2,4-Dichlorophenoxy acetic acid	58.8	576	[88]
Coconut shell	Slow pyrolysis at 700 °C (7 °C min^−1^, 2 h). Modified with H_3_PO_4_ (1 mol dm^−3^, BC3) or NaOH (1 mol dm^−3^, BC4); washed, dried (80 °C, 24 h), sieved <1 mm.	Diazinon	10.33	508	[89]
Sugarcane bagasse	Sun-dried (1 week), cut, pyrolyzed at 500 °C (10 °C min^−1^, 30 min, O_2_-limited).	Carbofuran	18.9	148.23	[90]
Palm Oil Fronds	Dried, carbonized at 700 °C (10 °C min^−1^, 2 h, N_2_ 150 cm^3^ min^−1^), impregnated with KOH (2.75:1), activated at 850 °C (N_2_/CO_2_, 1 h); washed (0.1 mol dm^−3^ HCl, H_2_O).	Carbofuran	164	1237.13	[91]
Lotus seedpod	Dried (85 °C, 24 h), sieved (<0.38 mm), impregnated with 85% H_3_PO_4_ (1:1–1:12, 24 h), pyrolyzed at 500–900 °C (10 °C min^−1^, 30–240 min); washed to neutral pH, dried (85 °C, 24 h), ground (<0.38 mm).	Metolcarb	12.6537	728.23	[92]
		Isoprocarb	12.9326		
		Pirimicarb	18.4554		
		Methiocarb	19.2862		
		Carbaryl	19.3357		
Nocino walnut	Walnut pomace dried (90 °C, 2 h; RT, 10 d), carbonized at 900 °C (N_2_, 5 °C min^−1^, 1 h). One sample was further treated with CO_2_ (100 L h^−1^, 1 h).	Chlorpyrifos	45.2	737	[17]

**Table 2:** Comparison of analytical methods for pesticide residue detection [103,104,105,106,107].

Method	Advantages	Limitations
Chromatographic techniques (GC–MS, LC–MS/MS)	Very high sensitivity and selectivity Capable of multi-residue analysis Widely accepted for regulatory compliance	Expensive instrumentation Requires trained personnel Time-consuming sample preparation Limited applicability in field settings
Spectroscopic methods (IR, Raman, UV-Vis)	Rapid and non-destructive Minimal sample preparation Suitable for high-throughput screening	Lower selectivity compared to chromatography Susceptible to matrix interferences Limited sensitivity for trace-level detection
Rapid/field methods (biosensors, immunoassays, test strips)	Portable and user-friendly Quick results (minutes) Useful for on-site monitoring in agriculture and food supply chains	Lower accuracy and reproducibility Limited detection range Stability issues under variable environmental conditions Often requires calibration

**Table 3:** Applications of chromatographic methods in pesticide residue detection across different food matrices.

Pesticide	Type of Food	Method for Detection	Linearity Range	LOD	LOQ	Recovery (%)	Reference
Atrazine and deltamethrin	Tomatoes, cucumbers, and brinjal	HPLC-UV-Vis	0.01–100.0 µg/L and 0.05–100 µg/L	0.01–0.05 µg/L	0.03 and 0.15 µg/L	80.9–98.6	[112]
Triazine	Water, tea, and juice samples	HPLC-DAD	1 –100 μg/L	0.3 μg/L	1 μg/L	80.1–90.6	[113]
Metolcarb, isoprocarb, and diethofencarb	Cabbage	HPLC-VWD	0.5 –100 ng/g	0.25–0.1 ng/g	-	92.4–99.6	[114]
Cyanazine, prometon, propazine, and prometryn	White gourd	HPLC-DAD	0.5–100 ng/g	0.1–0.2 ng/g	4.6–5.7 ng/g	80.3–120.6	[115]
Triazine	Potato, carrot, and lettuce	HPLC-DAD	0.0061–70 ng/mL	2.0–5.3 ng/mL	-	97.5–103.0	[116]
Epoxiconazole, flusilazole, tebuconazole, and triadimefon	Honey, mango, grape, and orange juice	HPLC-DAD	1–1000 µg/L	0.014–0.109 µg/L	0.047–0.365 µg/L	-	[117]
Triazine	Rice	HPLC-UV-Vis	-	0.010–0.080 µg/kg	-	83.9–103.5	[118]
Sulfometuron methyl, bensulfuron methyl, pyrazosulfuron ethyl, and chlorimuron ethyl	Pakchoi, spinach, and celery	HPLC-PDA	1 –150 μg/L	0.12–0.34 µg/L	-	87.1–108.9	[119]
Epoxiconazole, fenbuconazole, difenoconazole, thiabendazole, and pyraclostrobin	Lettuce	HPLC–MS	1.0–500 μg/L	0.25–1.0 µg/L	-	78.5–87.3	[120]
Triazine	Corn	HPLC–MS/MS	2.0–200.0 ng/g	0.01–0.12 ng/g	0.04–0.35 ng/g	73.37–107.37	[121]
Atrazine and prometryn	Pakchoi, lettuce, apple, pear, and strawberry	HPLC-PDA	0.5–200 ng/mL	0.18–0.72 ng/mL	-	88.0–101.9	[122]
Triazine	Rice	LC-MS	0.10 and 20 ng/g	1.08–18.10 pg/g	3.60–60.20 pg/g	79.3–116.7	[123]
Chlordane, heptachlor, lindane, and aldrin	Pepper	GC-ECD	0.05–100 ng/g	0.005–0.3 ng/g	0.017–0.1 ng/g	86.1–109.4	[124]
Phorate, diazinon, ethion, malathion, and fenthion	Apple, grape, pear, tomato, and green jujube	GC-FPD	0.05–100 μg/L	0.018 –0.045 µg/L	3–6 μg/L	84–116	[125]
Profenofos, phosalone, fenitrothion, and fenthion	Fruit juice	GC-FID	0.1 –100 ng/mL	0.03–0.21 ng/mL	-	91.9–99.5	[126]

DAD—diode array detector; VWD—variable wavelength detector; PDA—photodiode array detector; ECD—electron capture detector; FPD—flame photometric detector; FID—flame ionization detector; MS—mass spectrometry; MS/MS—tandem mass spectrometry.

**Table 4:** Spectroscopic methods for pesticide detection in food and water.

Pesticide	Type of Food	Method for Detection	Linearity Range	LOD	Recovery (%)	Reference
Methyl parathion	Chinese cabbage	SERS	-	1.26 µg/kg	-	[129]
Acephate	Rice samples	SERS	100.2–0.5 mg/L	0.5 mg/L	-	[130]
Chlorpyrifos	Tomato	SERS	10^−3^–10^−9^ mol/L	10^−9^ mol/L	-	[131]
Malathion	Strawberry	SERS	-	100 µg/kg	90–122	[132]
Cypermethrin	Kiwi	SERS	-	10^−10^ M	-	[133]
Atrazine	Apple juice	SERS	-	0.0012 mg/L	93	[134]
Phosmet, chlorpyrifos, and carbaryl	Orange, apple, and tomato	SERS	5–30 mg/L	2.94–6.66 µg/kg	-	[135]
Parathion	Cabbage washing solutions	SPR	0.01–1.84 mg/L	1.2 µg/L	86–114	[136]
Profenofos	Water	SPR	10^−4^–10^−1^ µg/L	2.5 × 10^−6^ µg/L	-	[137]
Triazophos	Cabbage, cucumber, and apple	SPR	0.98–8.29 ng/mL	0.096 ng/mL	84–109	[138]
Carbendazim	Medlar	SPR	0.05–150 ng/mL	0.44 ng/mL	102.4–115.0	[139]
Chlorpyrifos	Maize, apple, cabbage, and medlar	SPR	0.25–50.0 ng/mL	0.056 ng/mL	86.9–119.2	[140]

**Table 5:** Immunoassays for pesticide residue detection.

Pesticide	Type of Food	Method of Detection	LOD	IC50	Reference
Triazophos	Water and apple	ELISA	/	6.6 ng/mL	[141]
Carbaryl	Rice, maize, and wheat	ELISA	0.3 ng/mL	5.4 ng/mL	[142]
Carbofuran	Chinese cabbage, cucumber, and orange	Ic-ELISA	0.65 ng/mL	7.2 ng/mL	[143]
Triazophos	Water	Ic-ELISA	/	1.73 ng/mL	[144]
Cyantraniliprole and chlorantraniliprole	Bok choy	ELISA	1.2 ng/mL	1.5 ng/mL	[145]
Parathion	Chinese cabbage, cucumber, and lettuce	FEA	0.2 ng/mL	1.6 ng/mL	[146]
Carbaryl and carbofuran	Corn	paper-based immunoassay	0.02 and 60.2 ng/mL	0.8 ng/mL and 217.6 ng/mL	[147]

**Table 6:** Colorimetric and fluorescent (bio)sensors for pesticide residue detection.

Pesticide	Type of Food	Method for Detection	Linearity Range	LOD	Recovery (%)	Reference
Chlorpyrifos	Vegetable	Colorimetric sensor	0–25 mg/kg	8.6 mg/kg	-	[148]
Methyl-paraoxon and chlorpyrifos-oxon	Vegetable	Colorimetric sensor	0.1–0.9 µg/mL	5.3–18 ng/mL	95	[149]
Malathion	Tap water	Colorimetric biosensor	/	1.78 µg/mL	103	[150]
Malathion	Water	Colorimetric biosensor	/	60 ng/mL	80–106	[151]
Acetamiprid	Chinese cabbage, tomato, eggplant, and cucumber	Colorimetric biosensor	/	10 pM	87–105	[152]
Acetamiprid	Tea	Fluorescence biosensor	50–100 nM	3.2 nM	97.57–102.25	[153]
Acetamiprid	Honey and orange juice	Fluorescence biosensor	5 nM–1.2 μM	3 nM	94.9–104.2	[154]
Diazinon	Water and fruit juices	Fluorescence biosensor	0.5–500 nM	0.2 nM	95.07–101.90	[155]
Dichlorvos	Tomato and spinach	Fluorescent sensor	2.5–120 μg/L	1.0 μg/L	94.0–106.0	[156]
Dicofol	Tea	Fluorometric chemosensor	0–10 mg/L	200 μg/L	72.2–97.5	[157]
Ethoprophos	Tap water	Chemiluminescent biosensor	5–800 nM	1 nM	95.5–106.5	[158]
Dimethoate, dipterex, carbofuran, chlorpyrifos, and carbaryl	Water	Chemiluminescent biosensor	/	24 µg/mL	-	[159]

## Data Availability

No new data were created or analyzed in this study. Data sharing is not applicable to this article.
